# Treatment of Necrotic Teeth by Apical Revascularization: Meta-analysis

**DOI:** 10.1038/s41598-017-14412-x

**Published:** 2017-10-24

**Authors:** Ling He, Juan Zhong, Qimei Gong, Sahng G. Kim, Samuel J. Zeichner, Lusai Xiang, Ling Ye, Xuedong Zhou, Jinxuan Zheng, Yongxing Liu, Chenyu Guan, Bin Cheng, Junqi Ling, Jeremy J. Mao

**Affiliations:** 1 Columbia University Medical Center, Center for Craniofacial Regeneration 630W. 168 St, New York, NY 10032 USA; 2 Department of Operative Dentistry and Endodontics, Guanghua School of Stomatology, Hospital of Stomatology, Sun Yat-sen University, Guangzhou, Guangdong, 510055 China; 3 State Key Laboratory of Oral Diseases, West China Hospital of Stomatology, Sichuan University, Chengdu, Sichuan, China; 4 Columbia University College of Dental Medicine, Division of Oral and Maxillofacial Radiology, New York, NY, USA; 5 Columbia University College of Dental Medicine, Division of Endodontics, New York, NY, USA; 6 Columbia University Mailman School of Public Health, Department of Biostatistics, New York, NY, USA; 7 Department of Pathology and Cell Biology, Columbia University Physician and Surgeons, 630West 168th Street 15-402, New York, NY 10032 USA; 8 Department of Orthopaedic Surgery, Columbia University Physician and Surgeons, 630 West 168th Street PH10-102, New York, NY 10032 USA

## Abstract

Each year ~5.4 million children and adolescents in the United States suffer from dental infections, leading to pulp necrosis, arrested tooth-root development and tooth loss. Apical revascularization, adopted by the American Dental Association for its perceived ability to enable postoperative tooth-root growth, is being accepted worldwide. The objective of the present study is to perform a meta-analysis on apical revascularization. Literature search yielded 22 studies following PRISMA with pre-defined inclusion and exclusion criteria. Intraclass correlation coefficient was calculated to account for inter-examiner variation. Following apical revascularization with 6- to 66-month recalls, root apices remained open in 13.9% cases (types I), whereas apical calcification bridge formed in 47.2% (type II) and apical closure (type III) in 38.9% cases. Tooth-root lengths lacked significant postoperative gain among all subjects (*p* = 0.3472) or in subgroups. Root-dentin area showed significant increases in type III, but not in types I or II cases. Root apices narrowed significantly in types II and III, but not in type I patients. Thus, apical revascularization facilitates tooth-root development but lacks consistency in promoting root lengthening, widening or apical closure. Post-operative tooth-root development in immature permanent teeth represents a generalized challenge to regenerate diseased pediatric tissues that must grow to avoid organ defects.

## Introduction

Dental caries are among the most prevalent infectious diseases of the mankind^1–3^. Severe caries occur in ~21% of children and adolescents with immature permanent teeth^4^. Approximately 7% of deep caries in immature permanent teeth develop dental-pulp necrosis^5^. Furthermore, ~25% of children and adolescents experience dental trauma, and among them, ~27% contract pulp necrosis^6^. Together, ~5.4 million children and adolescents in the United States each year suffer from caries- and trauma-elicited pulp necrosis and tooth loss^7^. Upon metastasis, microbials and toxins of dental origin may cause systemic infections such as cavernous sinus thrombosis, cerebral abscess, myocardial infarctions and endocarditis^8,9^.

Necrotic immature permanent teeth in children and adolescents are a formidable clinical challenge^10^. Analogous to hepatitis-elicited liver growth arrest in children^11^, caries or trauma can induce necrosis of dental pulp and developmental arrest of immature permanent teeth^12^. Whereas infections of most organs are treated by antibiotics, dental-pulp infections are recalcitrant to systemic anti-microbial therapies due to early-onset necrosis. Upon necrosis, tooth survival not only relies on local infection control, but also restoration of dental-pulp vitality^13^. Local antimicrobial therapy alone, while effective in controlling pulp infections, fails to revert arrested tooth-root growth^13^. Apexification is the current treatment for pulp necrosis in children and adolescents by filling the disinfected root canal with inert materials, but leads to ~45.9% post-operative tooth fractures in nine years^14^. Following tooth loss in children and adolescents, dental implants are contraindicated because metallic implants are embedded in the growing alveolar bone in children whose alveolar bone undergoes active growth, for which metallic implants cannot adapt^15^. Thus, pulp necrosis at pediatric age is a pandemic without a clinically satisfactory therapy, either before or after tooth loss.

Regenerative therapies that enable the treated necrotic immature permanent teeth to complete root development have been tirelessly pursued. In 1961, Nygaard-Østby reported the first recognized study of evoked bleeding^16^. Histologic sections from the patient’s extracted teeth showed ingrowth of vascularized connective tissue in the root canal^16^. Subsequently, the practice of evoked bleeding in clinically treated necrotic immature teeth proliferated^14,17^. In 2011, the American Dental Association (ADA) issued clinical codes (D3351, D3352 and D3354) for the practice of evoked bleeding, also known as apical revascularization (AR)^13^. Presently, teaching of AR is incorporated in postgraduate endodontics training programs in the United States^8,9^. The European Society of Endodontology recently adopted AR^18^. Dental and/or endodontic societies in China and several other Asian countries are in the process of adopting AR.

To date, no meta-analysis has been reported on AR’s efficacy. Bose *et al*. (2009) performed a retrospective evaluation of radiographic outcomes in immature teeth with necrotic dental pulp treated with apical revascularization^5^, with a collection of 54 published and unpublished clinical cases but without performing PRISMA or meta-analysis. The primary goal of Bose *et al*. (2009) was to compare two root-canal disinfection protocols. The primary objective of the present study is to perform a patient-level meta-analysis of tooth-root development among all qualified AR cases in the literature using PRISMA with strict inclusion and exclusion criteria^19,20^. As opposed to the majority of tissue-engineering work with a focus on regenerating adult tissues, AR aims to regenerate pediatric tissues, and is not considered successful unless the treated immature teeth complete root development^12^. In general, whether and how injured or damaged pediatric tissues regenerate to heal defects is largely elusive^21,22^. Necrotic immature teeth offer powerful models in both experimental animals and human patients for devising effective therapies that regenerate growing, pediatric tissues to complete organ development.

## Results

**Figure 1:**
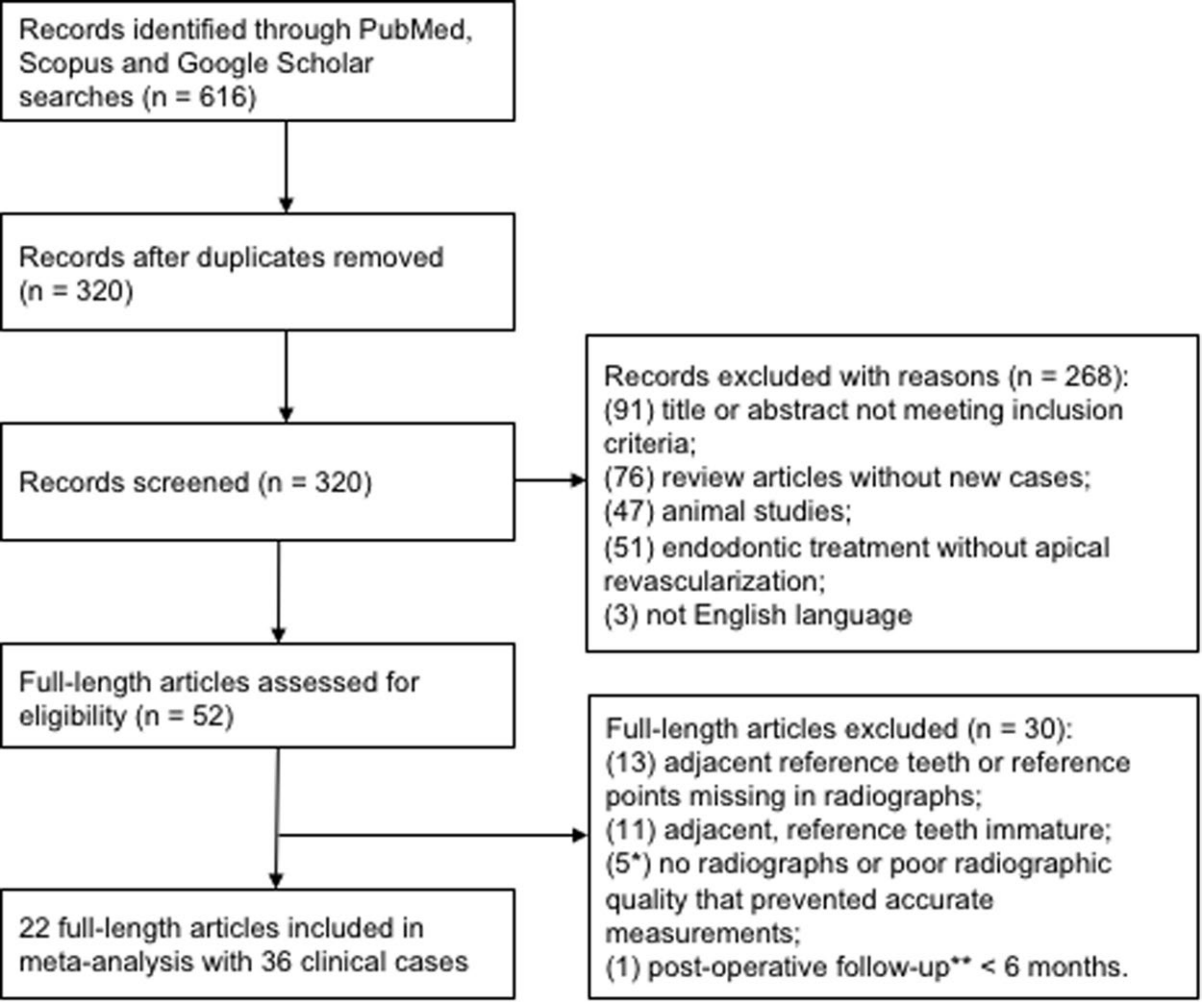
PRISMA flow chart. PRISMA guidelines were strictly followed^19,20^. Out of 616 records emerged, duplicates removed to yield 320 full-length publications. Cases were excluded using pre-defined inclusion and exclusion criteria in Table 1. Titles and abstracts of the 320 full-length studies without reviewing any data in each report’s Result section, and excluded 268 studies to yield the resulting 52 studies. The full text of the 52 full-length reports were carefully reviewed to further exclude 30 studies, with specific reasons as stated, to yield the final 22 studies included in meta-analysis with a total of 36 clinical cases.

Strict inclusion and exclusion criteria are shown in Table 1. The patients’ age range was 8-18 years old, reflective of the population with immature permanent teeth (Table 2). Although the included 36 cases were treated by clinicians from multiple continents including Americas, Asia, Australia, and Europe, disinfection and intra-canal medicament protocols were substantially similar (Table 2). Post-operative recalls ranged from 6 to 66 months (17.8 ± 11.6 months) (Table 2). Figure 1 shows PRISMA flow diagram. A total of 320 reports resulted from 616 hits following removal of duplicates. Next, a total of 268 studies were excluded: 91 with titles and abstract not meeting the inclusion criteria; 76 review articles; 47 non-human animal studies; 51 for treatment without AR performed; and 3 in non-English language (Fig. 1). Among the resulting 52 articles, 30 were further excluded: 13 without adjacent reference teeth or anatomic reference points; 11 with immature reference teeth; 5 without dental radiographs or poor radiographic quality and one remaining study with <6-month recall (Fig. 1). Thus, 22 full-length articles that fit the pre-defined inclusion criteria and were immune from the pre-defined exclusion criteria were selected for meta-analysis, with a total of 36 cases (Table 2). The Intraclass correlation coefficient (ICC) for the tooth length ratio, the optical width ratio, and the root area ratio were 0.85, 0.47, and 0.73, respectively, indicating that strong inter-examiner agreement for tooth length ratio and root area ratio but moderate for apical width ratio.

**Table 1:** Inclusion and exclusion criteria.

**Inclusion**
• Human clinical studies assessing the treatment effect of apical revascularization on necrotic immature permanent teeth
• Apical revascularization or evoked bleeding was the primary treatment protocol besides disinfection and antibiotics use
**Exclusion**
**1**. **Literature exclusion**
• The abstract and title do not meet the inclusion criteria Review articles without new cases
• Non-human animal studies
• *In vitro* studies
• Endodontic treatment without apical revascularization or evoked bleeding
• Non-English
**2. Clinical exclusion**
• No adjacent reference tooth or invisible reference anatomic locations
• No radiographic images provided or poor radiographic resolution*
• Post-operative follow-up < 6 months**
• Apical revascularization was performed in conjunction with orthodontic treatment

*Unable to measure the cemento-enamel junction (CEJ), the most prominent incisal edge or cuspal edge or root apex, despite effort made by at least four clinically qualified coauthors and further verified by an oral and maxillofacial radiologist (S.J.Z.). **Elimination of one case with the concern of unfair disadvantage for apical revascularization due to insufficient postoperative time. The majority of the clinical cases had more than 6-month recalls.

**Table 2:** Study IDs, patient demographics and self-reported peri-apical healing, apical closure, dental wall thickening and root lengthening.

Patient #	PMID	Authors, year	Journal	Age	Sex	Treated tooth #	Reference tooth #	Disinfection	Intracanal medication	Coronal barrier/ sealing	Follow-up (months)	Author reported			
												Peri-apical healing	Apical closure	Dentin wall thickening	Root lengthening
1	15085044	Banchs and Trope, 2004	J Endod	11	M	29	30	5·25% NaOCl, peridex	TAP	MTA, CR	6, 12, 18	Yes	Yes	Yes	Yes
2	18571000	Jung *et al*., 2008	J Endod	10	F	20	21	2·5% NaOCl	TAP	MTA, CR	12, 24	Yes	Yes	Yes	Yes
3				9	F	20	19				6, 24	Yes	Yes	Yes	Yes
4				14	F	29	28				12	Yes	NR	NR	NR
5	18634921	Shah *et al*., 2008	J Endod	16	F	10	9	2·5% NaOCl, 3% Hydrogen peroxide	FC	GIC	6, 24	Yes	Yes	Yes	Yes
6				16	F	9	8				6	Yes	Yes	Yes	Yes
7				12	F	8	9				6	Yes	Yes	Yes	Yes
8	19125982	Reynolds *et al*., 2009		11	F	20	19	6% NaOCl, 2%CHX	TAP	MTA, CR	18	Yes	NR	NR	Yes
9			Int Endod J	11	F	29	30				18	Yes	NR	NR	Yes
10	19410097	Ding *et al*., 2009	J Endod	8	M	9	8	5·25% NaOCl	TAP	MTA, CR	10, 15	Yes	Yes	Yes	NR
11	19912384	Shin *et al*., 2009	Int Endod J	12	F	29	30	6% NaOCl, 2% CHX	None	MTA, CR	7, 13, 19	Yes	Yes	Yes	Yes
12	20171379	Petrino *et al*., 2010	J Endod	11	M	29	30	5·25% NaOCl	TAP	MTA, CR	12	Yes	NR	Yes	Yes
13						20	19				12	Yes	NR	Yes	Yes
14	21133946	Thomson *et al*., 2010	Aust Dent J	12	F	20	21	1% NaOCl	TAP	MTA, GI, CR	18	Yes	NR	NR	Yes
15	22077958	Chen *et al*., 2012	Int Endod J	8	M	29	28	5·25% NaOCl	Ca(OH)_2_	MTA, CR	7, 13	Yes	Yes	Yes	NR
16				10	F	20	19				9	Yes	No	Yes	NR
17				10	M	20	19				7, 26	Yes	NR	Yes	NR
18	22627612	Kim *et al*., 2012	Int J Oral Sci	12	M	20	19	3% NaOCl	TAP	MTA, GP, CR	24	Yes	Yes	Yes	NR
19				10	M	29	28				42	Yes	Yes	Yes	NR
20	23146641	Jadhav *et al*., 2012	J Endod	18	F	9	8	2·5% NaOCl	TAP	GIC	6, 12	Yes	Yes	Yes	Yes
21				16	F	9	8				6, 12	Yes	Yes	Yes	Yes
22				21	M	8	7				6, 12	Yes	Yes	Yes	Yes
23				15	M	9	10				6, 12	Yes	Yes	Yes	Yes
24				23	M	8	9				6, 12	Yes	Yes	Yes	Yes
25	23880282	Shmizu *et al*., 2013	J Endod	9	M	9	8	2·6% NaOCl	Ca(OH)_2_	MTA, CR	12	Yes	NR	Yes	NR
26	24041394	Nosrat *et al*., 2013	J Endod	8	M	8	9	2·5% NaOCl	Augmentin	MTA, CR	17, 31	Yes	Yes	Yes	No
27	24332005	Becerra *et al*., 2014	J Endod	11	F	20	21	5·25% NaOCl	TAP	MTA, CR	12	Yes	Yes	Yes	No
28	25443280	Saoud *et al*., 2014	J Endod	11	M	8	9	2·5% NaOCl	TAP	MTA, CR	6, 9, 12	Yes	Yes	Yes	NR
29	25680956	Nevins *et al*., 2015	J Endod	14	F	13	12	6% NaOCl	DAP	GIC, CR	6, 12	Yes	Yes	NR	Yes
30	25684914	Narang *et al*., 2015	Contemp Clin Dent	*	*	9	8	2·5% NaOCl	TAP	GIC, CR	6, 18	NR	NR	NR	NR
31	25931029	Lei *et al*., 2015	J Endod	10	F	29	30	1% NaOCl	TAP	MTA, CR	6, 10	Yes	Yes	Yes	Yes
32	26587419	Park *et al*., 2015	Restor Dent Endod	20	F	29	30	5·25% NaOCl	TAP	MTA, CR	12	Yes	Yes	NR	No
33				10	F	29	30				12	Yes	Yes	NR	No
34	26647945	Miltiadous *et al*., 2015	Braz Dent J.	14	M	8	7	2·5% NaOCl	TAP	MTA, CR	24, 36	Yes	Yes	No	No
35	26884781	Khoshkhounejad *et al*., 2015	J Dent (Tehran)	16	M	9	8	5·25% NaOCl, 0·2%CHX	TAP	MTA, CR	6	Yes	NR	NR	NR
36	26949550	She *et al*., 2016	Case Rep Dent	12	M	4	5	3% NaOCl	Ca(OH)_2_	MTA, CR	7, 36, 66	Yes	Yes	Yes	NR

TAP: triple antibiotics paste; DAP: double antibiotics paste; GIC: glass ionomer cement; CR: composite resin; MTA: mineral trioxide aggregate; GP: gutta-percha; F: female; M: male; NR: not reported; Ca(OH)_2_: calcium hydroxide; NaOCl: sodium hypochlorite; FC: formocresol; CHX: chlorhexidine. *patients’ demographics provided as a group but not individually.

**Figure 2:**
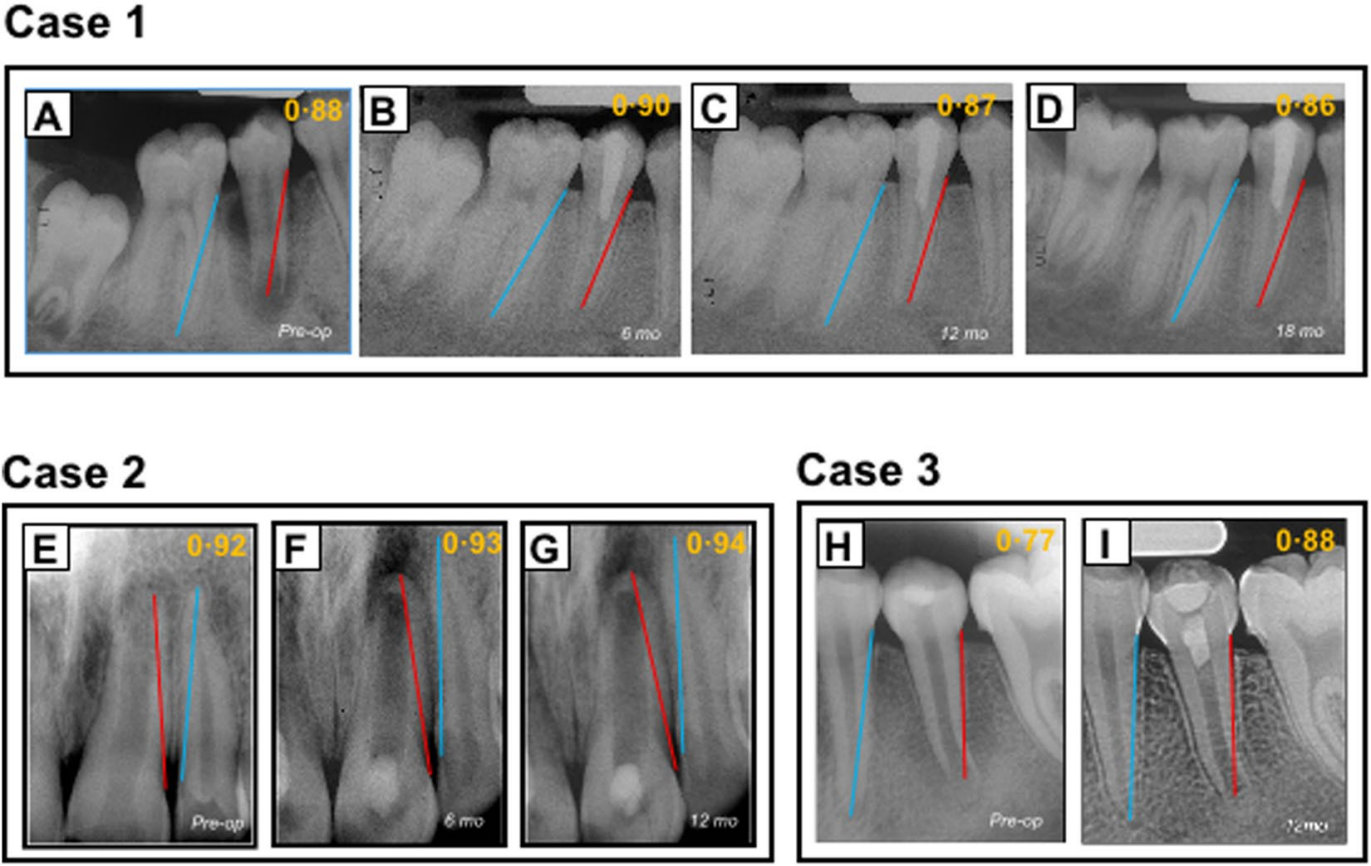
Illustrated clinical cases of apical revascularization. Three cases illustrate not only representative clinical outcome and its diversity, but also intrinsic radiographic image distortion. The linear tooth-root length of the treated tooth (red lines); root lengths of the reference tooth (blue lines). In Case 1 (PMID 15085044), apical revascularization performed in the second mandibular premolar (P2) of an 11-year-old male patient (**A**), with 6-, 12- and 18-month recalls (**B**,**C**,**D**, respectively). Root-length ratios: 0.88, 0.90, 0.87 and 0.86. Apical closure at 18-month recall (**D**). Apical radiolucency resolved at 6-, 12- and 18-month recalls. In Case 2 (PMID 23146641), a necrotic central incisor of a 16-year-old female patient with arrested root-apex development (**E**) treated with apical revascularization and recalls at 6- and 12-months (F,G, respectively) with root-length ratios at 0.92, 0.93 and 0.94. Apical calcification bridge at both 6- and 12-month recalls (**F**,**G**). Modest apical radiolucency before treatment (**E**), but apparently became pronounced at 6- and 12-month recalls (**F**,**G**). In Case 3 (PMID 24332005), a necrotic second mandibular premolar of an 11-year-old female patient with arrested root-apex development (**H**) treated with apical revascularization, and 12-month recall (**I**). Root-length ratios at 0.77 to 0.88. No apparent apical closure at 12-month recall (**I**). Substantial pre-treatment apical radiolucency (**H**) resolved at 12 months (**I**).

**Figure 3:**
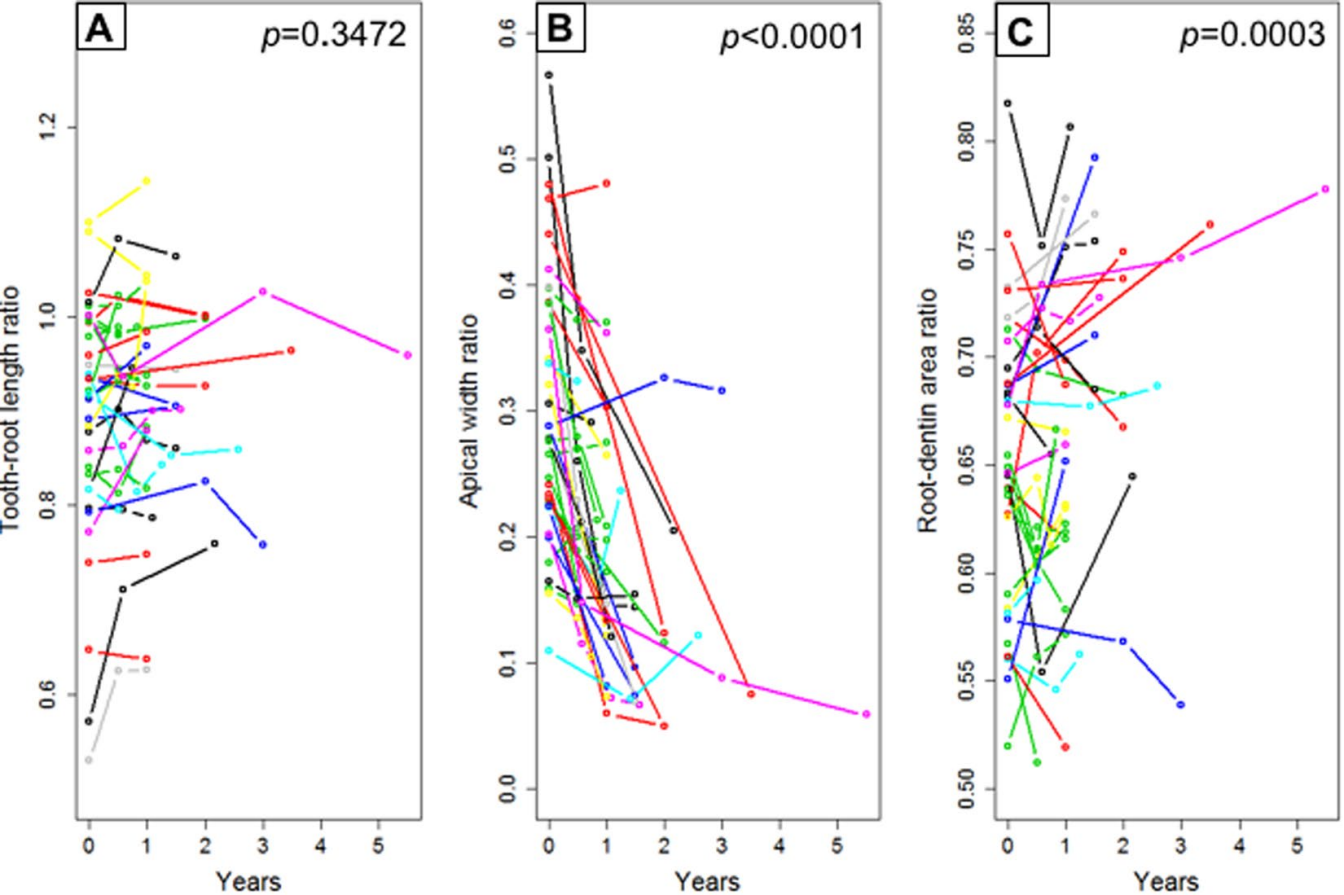
Statistical analysis of tooth-root development following apical revascularization. (**A**) Tooth-root length ratios of all 36 included clinical cases revealing no significant postoperative root lengthening (*p* = 0.3472). (**B**) Apical width ratios of all 36 included clinical cases revealing significant postoperative narrowing of root apices (*p* < 0.0001), but this significance is restricted to types II and III cases, not type I patients (c.f. Supplemental Fig. 2B). (**C**) Root-dentin area ratios of all 36 included clinical cases revealing significant increase in postoperative root-dentin area (*p* = 0.0003), but this significance is restricted to types III cases, not type I and II patients (c.f. Supplemental Fig. 3B).

Tooth-root lengths and root/crown ratios are of paramount importance to fracture rates and hence tooth loss^23^. Three cases were demonstrated in Fig. 2, which were selected from the included 36 patients to illustrate not only representative clinical outcome of tooth-root development and its diversity following AR, but also intrinsic radiographic image distortion. In Case 1 (PMID 15085044, corresponding to Patient #1 in Table 2), AR was performed in the necrotic second mandibular premolar of an 11-year-old male (Table 2). The absolute tooth-root length (red line) indeed increased at 6-, 12- and 18-month recalls, so was root length of the reference tooth (blue line) also increased (Supplemental Clinical Data 1: Patient 1), with virtually no increases in root-length ratios: 0.88, 0.90, 0.87 and 0.86 (Fig. 2A–D), suggesting little increase in root length following AR. Apical closure occurred at 18 months (Fig. 2D), suggesting that apical closure is not necessarily associated with root lengthening. Apical radiolucency was resolved at 6-, 12- and 18-month recalls (Fig. 2B–D). Case 2 (PMID 23146641, corresponding to Patient #21 in Table 2) represents a necrotic central incisor of a 16-year-old female (Fig. 2E). The linear tooth-root lengths of the treated tooth (red line) indeed increased at 6- and 12-month recalls, but so were the root lengths of the reference tooth (blue line) (Supplemental Clinical Data 1: Patient 21) (Fig. 2F,G). Accordingly, root-length ratios showed no substantial increases: 0.92, 0.93 and 0.94 for pre-treatment and 6- and 12-month recalls (Fig. 2E–G), suggesting little gain in root lengths. Apical calcification bridge (ACB) was present at both 6- and 12-month recalls (Fig. 2F,G). Apical radiolucency was modest before treatment (Fig. 2E), but became pronounced at 6- and 12-month recalls (Fig. 2F,G). Case 3 (PMID 24332005, corresponding to Patient #27 in Table 2) is a necrotic second mandibular premolar of an 11-year-old female (Fig. 2H). The root-length ratios were 0.77 pre-treatment and 0.88 by the 12-month recall (Fig. 2I), indicative of root lengthening. Remarkably, there was no apical closure, suggesting that apical closure and root lengthening are not necessarily associated with each other. Substantial pre-treatment apical radiolucency was resolved at 12 months (Fig. 2I). Table 3 provides root-length ratios of all 36 included cases. Statistical analysis of root-length ratios of all 36 cases lacked post-operative gain (*p* = 0.3472) (Fig. 3A), contrasting to self-reported 52.8% root lengthening in 19 of the 36 cases (Supplemental Table 1). Plots of root-length ratios of 36 cases are shown in Supplemental Fig. 1A. Radiographic images of all 36 included clinical cases with and without current tooth-length measurements are provided in Supplemental Clinical Data 1 and 2 for any interest in verifying data validity and reproducibility.

**Table 3:** Ratios of tooth-root length, apical width and root-dentin area before and after apical revascularization.

Patient#	PMID	Recall (months)	Tooth-root length ratio	Apical width ratio	Root-dentin area ratio
1	15085044	0	0·88	0·50	0·68
		6	0·90	0·26	0·72
		12	0·87	0·15	0·75
		18	0·86	0·14	0·75
2	18571000	0	0·93	0·24	0·72
		12	0·93	0·06	0·70
		24	0·93	0·05	0·67
3	18571000	0	0·99	0·48	0·63
		6	1·02	0·39	0·70
		24	1·00	0·12	0·75
4	18571000	0	0·74	0·39	0·64
		12	0·75	0·30	0·62
5	18634921	0	1·00	0·18	0·71
		6	0·98	0·21	0·69
		24	1·00	0·12	0·68
6	18634921	0	0·92	0·28	0·57
		6	1·02	0·16	0·51
7	18634921	0	0·98	0·16	0·64
		6	0·99	0·15	0·61
8	19125982	0	0·94	0·20	0·69
		18	0·91	0·07	0·71
9	19125982	0	0·89	0·29	0·68
		18	0·90	0·10	0·79
10	19410097	0	0·94	0·22	0·56
		10	0·81	0·16	0·55
		15	0·84	0·24	0·56
11	19912384	0	0·86	0·20	0·71
		7	0·86	0·11	0·72
		13	0·90	0·07	0·72
		19	0·90	0·07	0·73
12	20171379	0	1·09	0·32	0·67
		12	1·04	0·12	0·67
13	20171379	0	1·10	0·34	0·58
		12	1·14	0·26	0·63
14	21133946	0	0·95	0·28	0·73
		18	0·94	0·07	0·77
15	22077958	0	0·80	0·28	0·82
		7	0·80	0·21	0·75
		13	0·79	0·12	0·81
16	22077958	0	0·82	0·31	0·68
		9	0·95	0·29	0·66
17	22077958	0	0·57	0·57	0·64
		7	0·71	0·35	0·55
		26	0·76	0·20	0·64
18	22627612	0	1·03	0·23	0·73
		24	1·00	0·05	0·74
19	22627612	0	0·93	0·44	0·69
		42	0·96	0·07	0·76
20	23146641	0	0·84	0·28	0·52
		6	0·81	0·28	0·56
		12	0·88	0·21	0·57
21	23146641	0	0·92	0·25	0·65
		6	0·93	0·19	0·60
		12	0·94	0·17	0·62
22	23146641	0	0·83	0·27	0·63
		6	0·84	0·27	0·62
		12	0·82	0·27	0·62
23	23146641	0	1·00	0·23	0·64
		6	0·94	0·20	0·60
		12	0·93	0·20	0·62
24	23146641	0	1·01	0·04	0·59
		6	1·01	0·37	0·61
		12	1·04	0·37	0·58
25	23880282	0	0·91	0·22	0·55
		12	0·97	0·08	0·65
26	24041394	0	0·92	0·11	0·68
		17	0·85	0·07	0·68
		31	0·86	0·12	0·69
27	24332005	0	0·77	0·41	0·65
		12	0·88	0·36	0·66
28	25443280	0	0·88	0·15	0·63
		6	0·94	0·14	0·64
		9	0·93	0·10	0·61
		12	1·04	0·07	0·63
29	25680956	0	0·53	0·40	0·72
		6	0·62	0·23	0·73
		12	0·63	0·15	0·77
30	25684914	0	1·01	0·16	0·69
		6	1·08	0·15	0·71
		18	1·06	0·15	0·68
31	25931029	0	0·99	0·38	0·65
		6	0·98	0·27	0·61
		10	0·99	0·21	0·67
32	26587419	0	0·96	0·23	0·76
		12	0·98	0·13	0·69
33	26587419	0	0·65	0·47	0·56
		12	0·64	0·48	0·52
34	26647945	0	0·79	0·29	0·58
		24	0·82	0·33	0·57
		36	0·76	0·32	0·54
35	26884781	0	0·82	0·34	0·58
		6	0·80	0·32	0·60
36	26949550	0	1·00	0·36	0·68
		7	0·94	0·15	0·73
		36	1·03	0·09	0·75
		66	0·96	0·06	0·78

**Figure 4:**
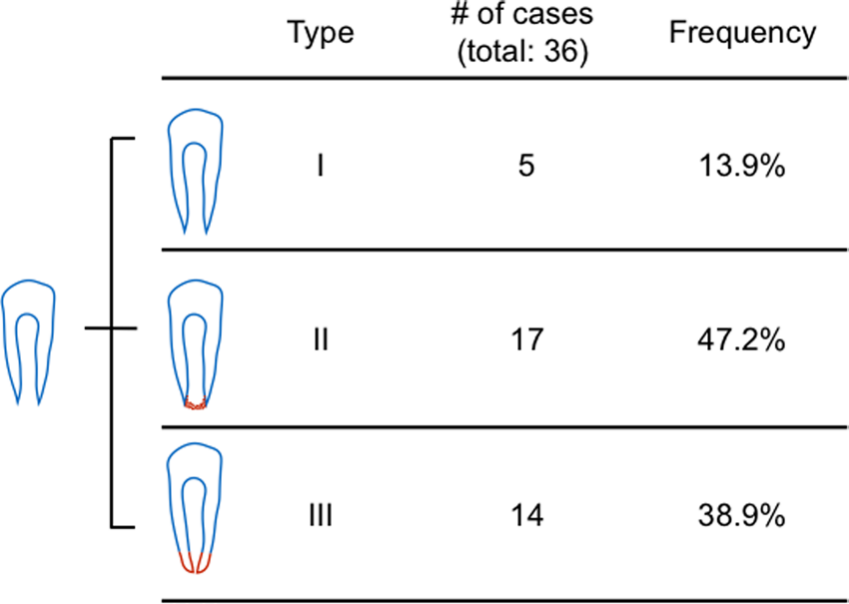
Three types of apical development following apical revascularization. We divided root development of immature permanent teeth into three subgroups following therapeutic intervention, such as apical revascularization. Type I represents little or no postoperative apical narrowing, with open apices comparable to pre-intervention. Type I occurred in 5 of the included 36 cases at 13.9% (top diagram; also c.f. Supplemental Movie 1A). Type II represents apical calcification bridge formation, and occurred among 17 of the included 36 cases at 47.2% (middle diagram; also c.f. Supplemental Movie 1B). Type III represents apical closure to a degree similar to a fully mature tooth that has completed root development, and occurred among 14 of the included 36 cases at 38.9% (bottom diagram; also c.f. Supplemental Movie 1C).

Another important indication of tooth-root development is apical closure, or lack thereof, because immature permanent teeth without apical closure are 2.75 times more likely to fracture than fully mature teeth^24^. Apical closure status of post-operative immature permanent teeth was divided into three categories in reference to Moorrees stages of normal root development^25^. Type I occurred in 5 out of 36 cases at 13.9% (Fig. 4 top row), with open apices comparable to pre-intervention (Supplemental Movie 1A), as in the demonstrated Case 3 in Fig. 2H,I. Type II occurred in 17 cases and consisted of the majority of the included 36 cases at 47.2% (Fig. 4 middle row), with ACB formation (Supplemental Movie 1B), as the demonstrated Case 2 in Fig. 2E–G. Type III occurred in 14 out of 36 cases at 38.9% with apical closure (Fig. 4 bottom row, and Supplemental Movie 1C), as the demonstrated Case 1 in Fig. 2A–D. Contrastingly, only one out of the total 36 cases was self-reported as no apical closure at 2.8% (Supplemental Table 2). Apical closure was self-reported in 25 cases at 69.4%, along with no self-reporting on apical closure status in 10 cases at 27.8% (Supplemental Table 2). To resolve this discrepancy between self-reported apical closure status (Supplemental Table 2) and the present evaluation (Fig. 4), apical-width ratios were measured by dividing the linear apical-opening width against tooth-crown width at CEJ. Relatively high fidelity emerged in apical-width ratios (Supplemental Fig. 2A) with author-graded apical closure status (Fig. 4). Type I cases showed no significant apical narrowing (*p* = 0.1568), but apical narrowing was present in both types II cases (*p* = 0.0002; slope: −0.00261) and III cases (*p* < 0.0001; slope: −0.00655) (Supplemental Fig. 2B).

Tooth-root lengthening was further analyzed among types I, II and III subgroups and again found no significant post-operative root-length gain in any subgroup: *p* = 0.1546 for type I, *p* = 0.4981 for type II, and *p* = 0.2439 for type III (Supplemental Fig. 1B). The average root-dentin area ratio showed significant post-operative gain (*p* = 0.0003), but this gain was restricted to type III apical closure cases (*p* < 0.0001), with no significant gain in root-dentin area ratio among type I or II cases (Supplemental Fig. 3B). Type II was associated with a significant decrease in root-dentin area ratio (*p* = 0.0229) (Supplementary Fig. 3B), likely due to enlarged root-canal space following curved ACB formation. Root-area ratios showed high fidelity in differentiating type III from types I and II cases. Per receiver operating characteristic (ROC) analysis, type I was associated with root-area ratios < 0.58, type II between 0.58 and 0.79, and type III >  0.79.

Apical radiolucency in patients with necrotic dental pulp suggests metastasis of microbial infections to the peri-apical space. Peri-apical infections are severe clinical conditions, and if left untreated, may cause systemic infections^8,9^. With a uniform standard and by stratifying apical radiolucency into four categories, apical radiolucency indeed was resolved in 26 out of the total 36 cases at 72.2% (Supplemental Table 3). However, apical radiolucency remained present in 8 cases at 22.2% (Supplemental Table 3). Two cases were free from pre-treatment and post-operative apical radiolucency, accounting for 5.6% (Supplemental Table 3).

## Discussion

Coagulated blood is perceived to harbor factors that heal tissue defects^26,27^. As a clinician initiated “regenerative” procedure, AR is analogous to other blood-clotting therapies such as microfracture in orthopedics where blood from bone marrow is induced to coagulate in focal articular cartilage defects for cartilage regeneration^28^. Can the same circulating blood induce the regeneration of different tissues such as articular cartilage and mineralized dentin? The intuitive answer might be that coagulated blood only serves as a scaffold, and the local environment determines what tissue is regenerated. However, local regenerative cues likely are insufficient to heal the defect in the first place, or otherwise there is no defect. What blood components, if any, activate local cues to regenerate specific tissues warrants experimental studies. Bioactive cues, local and/or blood-derived, must form a chemotactic gradient to recruit cells for tissue regeneration. Whether coagulated blood yields a chemotactic gradient lacks experimental evidence, and should be investigated.

For AR or any other regenerative therapies to succeed in enabling post-operative tooth-root development in children and adolescents, tooth roots must undergo axial growth (lengthening), transverse growth (dentin-wall thickening) and convergence growth (apical closure) (Supplemental Movie 2). A lack of significant tooth-root lengthening in the present study is to our surprise and at variation with 52.8% self-reported root lengthening among the included 36 cases. The present finding of a lack of root-lengthening following AR is directly supported by absence of tooth-length increase among 34 trauma cases of immature permanent teeth by comparing AR with Apexification by cone-beam CT measurements^37^. Intuitively, root lengthening is necessary for apical closure. However, the present data only support root-dentin wall thickening, but not root lengthening, even in type III apical-closure cases. The average absolute tooth-root length increased by 1.2 millimeters in 18 months following AR^29^, perhaps too small to be clinically meaningful and may be susceptible to intrinsic measurement errors. A lack of significant tooth-root lengthening following AR is probably attributed to the observation that apical calcification bridge^30^, present in 47.2% of all included AR cases as the largest subgroup, may limit axial root lengthening. We speculate that apical calcification bridge (ACB) formation, perceived as a bridge in 2D radiographs, is actually a band of transverse mineralization in 3D, and may not develop into a pointy and closed apex. ACB differs from apical closure as a distinctive subgroup, as confirmed by a lack of root-dentin widening in the ACB subgroup but increased root-dentin area in the apical-closure subgroup. Post-operative root lengths in individual patients may indeed increase as shown in Case 3. However, a substantial root-length gain of AR-treated necrotic immature permanent teeth in children and adolescents as a population may not be a realistic expectation. Ratios of root lengths, apical widths and root-dentin areas were used in the present study to account for radiographic image distortion that is unavoidable among radiographs taken over time and by multiple practitioners^31^.

The present study is limited to the teaching of clinical cases included, in absence of any prospective and randomized clinical trial on AR’s efficacy. In experimental animal studies and several clinical case reports in which the teeth were extracted following AR, ingrowth of vascularized soft connective tissue was present in the treated root canal^32,33^. However, there is remarkable variation regarding the frequency, amount and nature of AR-induced tissue ingrowth^32,34^, potentially accounting for the diversity of types I, II and III outcome of the included clinical cases. Together, post-operative tooth-root development represents a general challenge to regenerate pediatric tissues that must grow to avoid organ defects^21,22^. Whether circulating blood activates local molecular cues to regenerate different and specific tissues warrant experimental and clinical investigations.

## Methods

### Search strategy

PubMed, Scopus and Google-Scholar searches were performed using ((revascularization OR revitalization OR (regenerative endodontics) OR (regeneration)) AND (immature teeth) from inception of each database to September 15, 2016.

### Study selection: PRISMA, inclusion and exclusion criteria

Transparent Reporting of Systematic Reviews and Meta-analyse (PRISMA) was adhered in study selection (Fig. 1). Inclusion and exclusion criteria were pre-defined (Table 1). As in Fig. 1, cases were excluded, for example, because the cemento-enamel junction (CEJ) or root apex was unrecognizable on radiographs despite independent effort made by at least four clinically qualified coauthors, and further verification by an oral and maxillofacial radiologist (S.J.Z.). Three coauthors independently screened the titles and abstracts of the 320 full-length studies without reviewing any data in each report’s Result section, and excluded 268 studies (Fig. 1) strictly based on the pre-defined exclusion criteria (Table 1) to yield the resulting 52 studies. Subsequently, three coauthors carefully read the full texts of the 52 full-length reports and further excluded 30 studies, with specific reasons stated in Results below (also c.f. Fig. 1).

### Apical revascularization

The following generic AR treatments were representative among the included studies (Table 2). Following rubber-dam isolation, local anesthesia and tooth access preparation, necrotic root canals were disinfected using irrigants such as sodium hypochlorite and chlorhexidine at the first visit. Calcium hydroxide or antibiotic pastes were applied into the canals as inter-appointment medicaments (Table 2). At the second visit, root canals were cleaned with irrigants to remove the intracanal medicaments. Apical bleeding was induced by passing a hand instrument beyond the apical foramen to allow blood filling in the canal. Mineral trioxide aggregate was placed directly over the coagulated blood clot. The coronal access was sealed with permanent restorations.

### Quantitative measurements of tooth-root development

To minimize potential bias, radiographs of all 36 cases were assigned to two clinically qualified coauthors with randomized and blinded radiograph sequence. Tooth-root development was quantified digitally on pre- and post-operative radiographs: root lengths as ratios of the treated and adjacent reference teeth, root-dentin geometry by subtracting root-canal area from tooth-root area, and apical closure as the ratio of crown and apical opening widths. The linear tooth-root length was measured independently by the two examiners from the CEJ to root apex digitally on all pre- and post-operative radiographs per existing methods^31,35^. To compensate for potential radiographic distortion with images taken over time and by multiple practitioners in the literature, the linear root length of not only the treated teeth (TT), but also the adjacent reference teeth (RT) were measured as long as the reference teeth had already completed root development^31^. TT/RT ratios were calculated not only to minimize radiographic image distortion, but also for cross-patient comparisons^31^. For immature permanent teeth with open apices, a linear line was drawn to connect apical tips with the center of the transverse line used as the apical end for root-length measurements^31^. Apical calcification bridge^30^ was measured as a transverse mineralization band that joined apical root tips^30,31^ blindly and independently by the two examiners. Root-dentin area was measured by subtracting root-canal area from the total root area that was defined from the apex to a transverse line connecting two CEJs^36^. Root-dentin area ratios were calculated by dividing root-dentin area against the total root area. Apical-width ratios were calculated by dividing apical-opening width against the width of a transverse line connecting the two CEJs per tooth. Apical radiolucency was stratified into four categories: −/−: no apical radiolucency before or after treatment; −/+: no apical radiolucency before treatment but detected apical radiolucency following treatment; +/+: apical radiolucency both before and after treatment; +/−: apical radiolucency before treatment but resolved following treatment.

### Statistical analysis

Random effects approach was adopted in absence of consistent measure of tooth-root development among the included 22 studies. The meta-analysis investigated whether tooth-root length ratios, apical width ratios, and root area ratios had any significant change following AR using a linear mixed effects model where recall time was the main predictor, controlling for the value at baseline, and the type of the treated tooth (e.g. incisors vs. premolars) if it proved significant. Random effects were included in the model to account for variations among the included 22 studies. Nested random effects within each study were included to account for variations among subjects within each study, with additional random effects included to account for variations between the two independent examiners. Intraclass correlation coefficient (ICC) was used to evaluate the inter-examiner reliability of the three outcomes. We further investigated whether tooth-root length ratios, apical width ratios, and root area ratios differed significantly among the three subgroups of apical closure status by assessing the moderating effect of apical closure type. For any significant post-operative change, cutoff values were determined by the receiver operating characteristic (ROC) method for apical closure prediction. All statistical analysis was conducted using SAS version 9.4, and plots were generated using R version 3.0.1. A p-value < 0.05 was considered as statistically significant.

## Electronic supplementary material

Supplementary material

Supplemental movie 1

Supplemental movie 2
