# Supplementary material for: Treatment of Necrotic Teeth by Apical Revascularization: Meta-analysis
Source: Sci Rep. 2017 Oct 24;7:13941. doi: 10.1038/s41598-017-14412-x (PMC5655000; doi:10.1038/s41598-017-14412-x)

Appendix

Treatment of Necrotic Teeth by Apical Revascularization: Meta-analysis

Ling He, DDS1,2+, Juan Zhong, DDS1,3+, Qimei Gong, DDS, PhD1,2+, Sahng G. Kim, DDS5, Samuel J. Zeichner, DDS4, Lusai Xiang, DDS, PhD1,2, Ling Ye, DDS, PhD3, Xuedong Zhou, DDS, PhD3, Jinxuan Zheng, DDS1,2, Yongxing Liu, PhD2, Chenyu Guan, DDS2, Bin Cheng, PhD6*, Junqi Ling, DDS, PhD2*, Jeremy J. Mao, DDS, PhD1,4,7,8*

Contents:

Suppl. Table 1: Self-reported root lengthening, or lack thereof, following apical revascularization…………………2

Suppl. Table 2: Self-reported post-operative apical closure, or lack thereof, following apical revascularization…….3

Suppl. Table 3: Self-reported peri-apical radiolucency, or lack thereof, following apical revascularization, and our assessment…………………….………………………………………………………………....4

Suppl. Figure 1A: Plots of tooth-root length ratios of all included apical revascularization cases……….………..5

Suppl. Figure 1B: Tooth-root length ratios plotted by author-graded apical closure types…………………..……..6

Suppl. Figure 2A: Plots of apical width length ratios of all included apical revascularization cases………….…...7

Suppl. Figure 2B: Apical width ratios plotted by author-graded apical closure types………………………………..8

Suppl. Figure 3A: Plots of root-dentin area ratios of all included apical revascularization cases……………….…9

Suppl. Figure 3B: Root-dentin area ratios plotted by author-graded apical closure types…………………………..10

Supplemental Table 1. Self-reported root lengthening, or lack thereof, following apical revascularization.

	# of cases (total: 36)	%
**Root lengthening**	19	52·8%
**Not reported**	12	33·3%
**No root lengthening**	5	13·9%

Supplemental Table 2. Self-reported post-operative apical closure, or lack thereof, following apical revascularization.

	# of cases (total: 36)	%
**Apical closure**	25	69·4%
**Not reported**	10	27·8%
**No apical closure**	1	2·8%

Supplemental Table 3. Self-reported peri-apical radiolucency, or lack thereof, following apical revascularization, and our assessment.

Self-reported			Our assessment		
**End-point PAR**	**# of cases (total: 36)**	**%**	**Pre-op/End-point PAR**	**# of cases (total: 36)**	**%**
-	35	97·2%	-/-	2	5·6%
			-/+	0	0%
+	1	2·8%	+/+	8	22·2%
			+/-	26	72·2%

PAR: peri-apical radiolucency; +: apical radiolucency present. –: apical radiolucency absent.

Supplemental Figure 1A.

Plots of tooth-root length ratios of all included apical revascularization cases.

Plots of tooth-root length ratios of all 36 cases in 22 included studies. Several studies each reported multiple clinical cases.

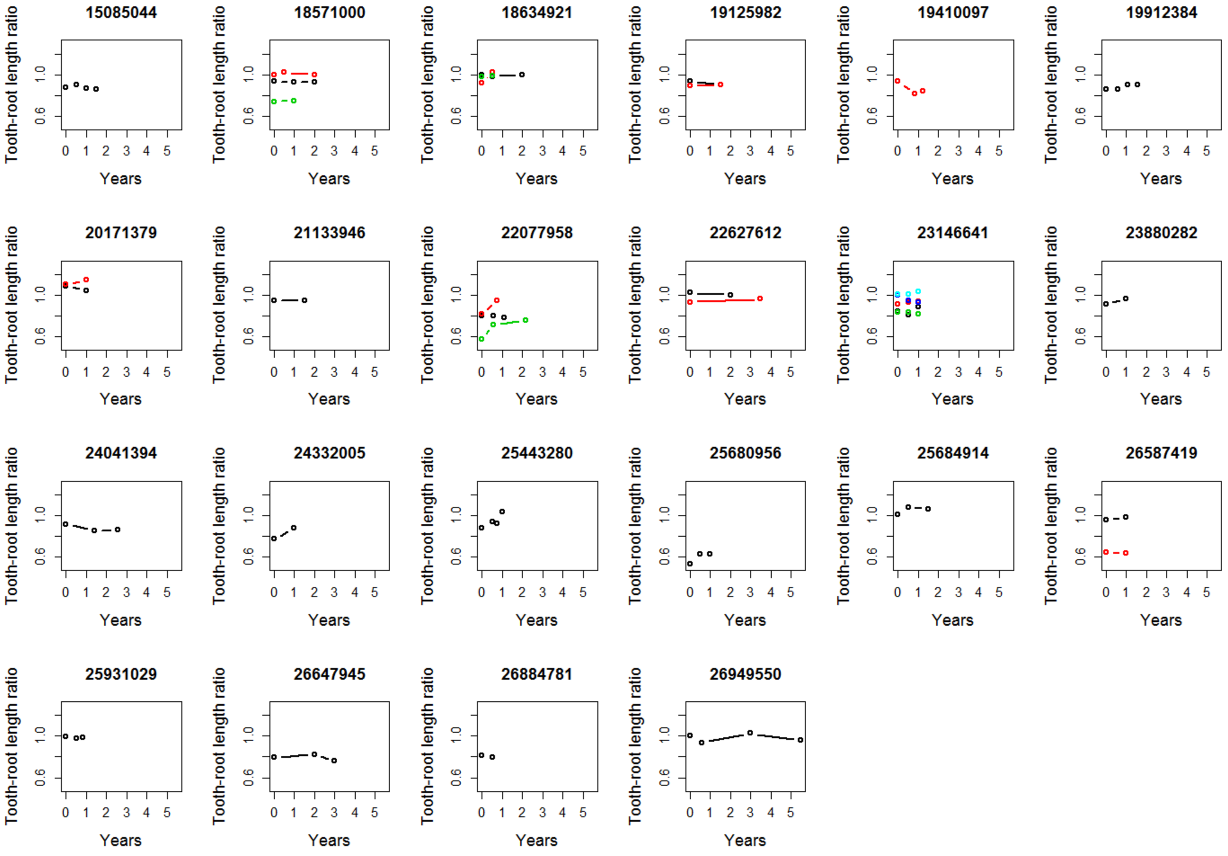

Supplemental Figure 1B.

Tooth-root length ratios plotted by author-graded apical closure types.

Tooth-root length ratios were calculated by dividing the length of treated teeth against the length of reference teeth by measuring from the apex to either CEJ or crown edge/cusp. Individual lines keyed to patients in Fig. 3.

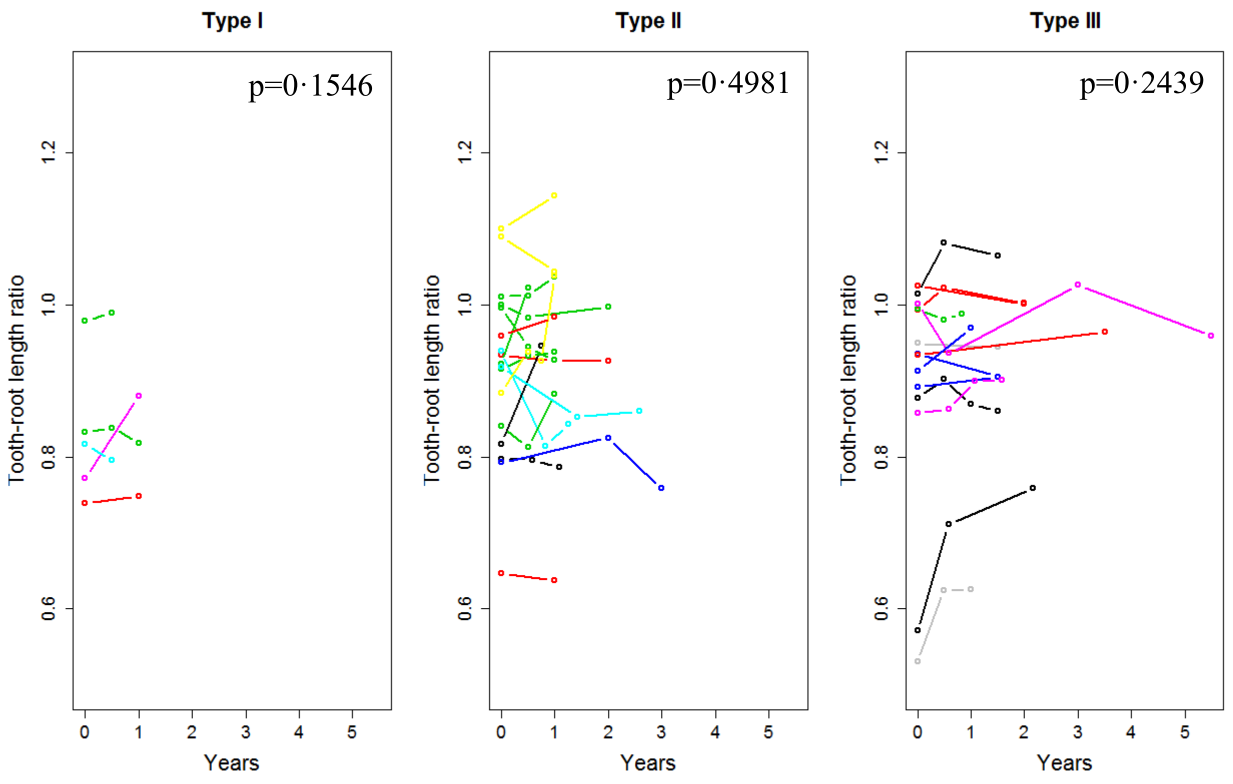

Supplemental Figure 2A.

Plots of apical width ratios of all included apical revascularization cases.

Plots of tooth-root length ratios of all 36 cases in 22 included studies. Several studies each reported multiple clinical cases.

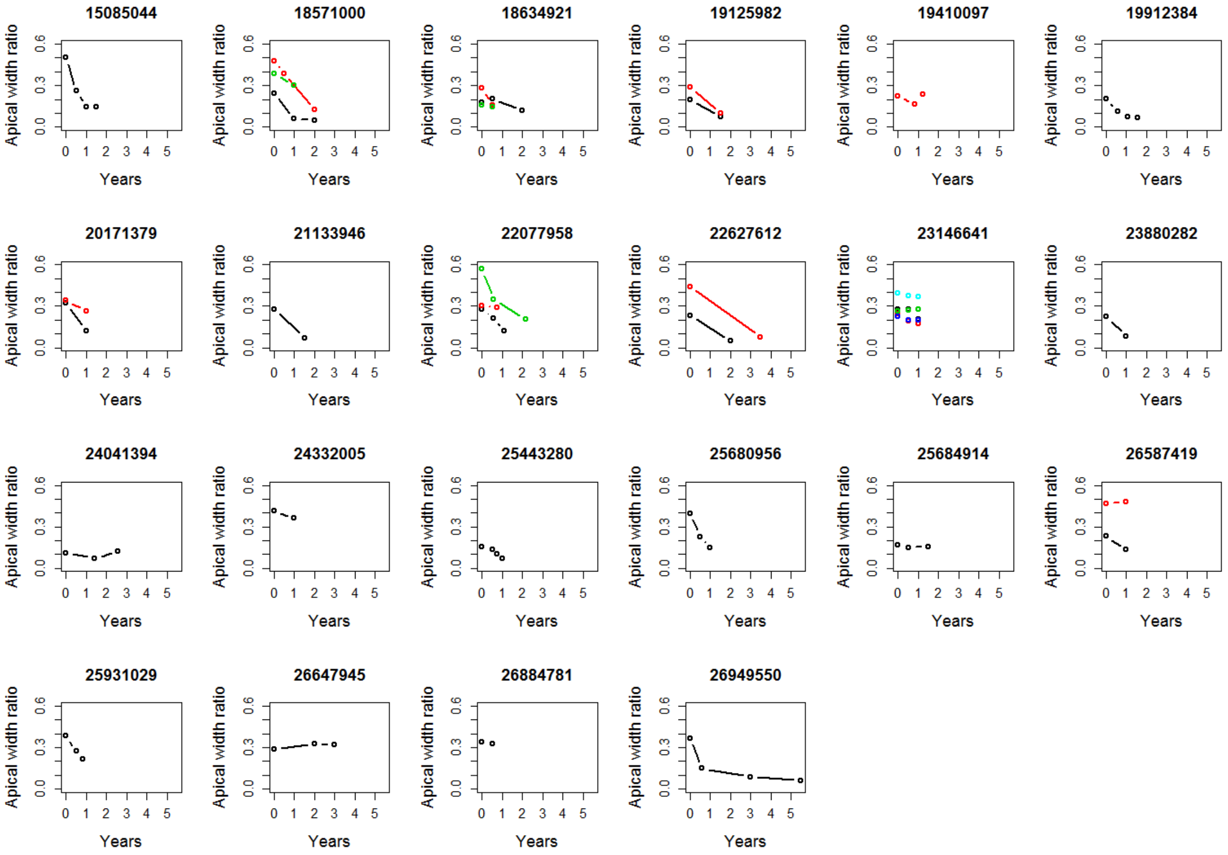

Supplemental Figure 2B.

Apical width ratios plotted by author-graded apical closure types.

Apical width ratios measured as the ratio of pre- and post-operative apical-opening width and a transverse line connecting the CEJs per tooth. Individual lines keyed to patients in Fig. 3.

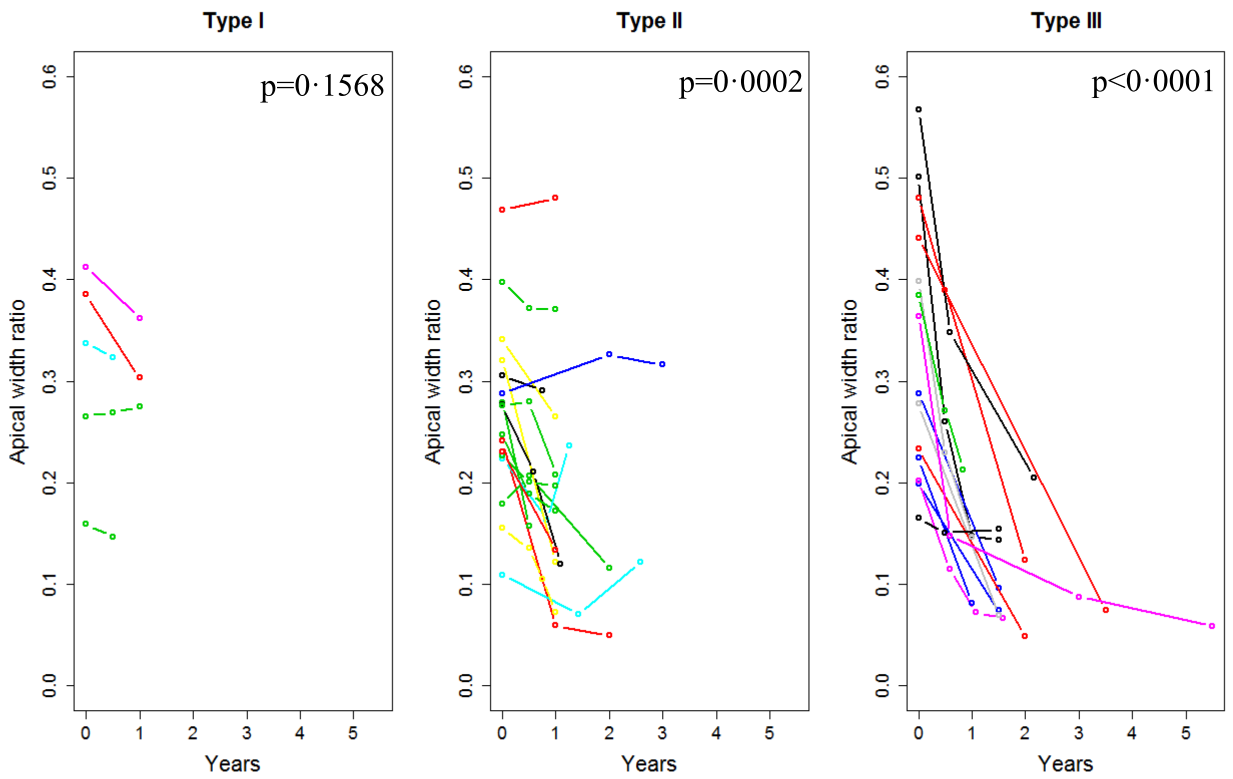

Supplemental Figure 3A.

Plots of root-dentin area ratios of all included apical revascularization cases.

Plots of root-dentin area ratios of all 36 cases in 22 included studies. Several studies each reported multiple clinical cases.

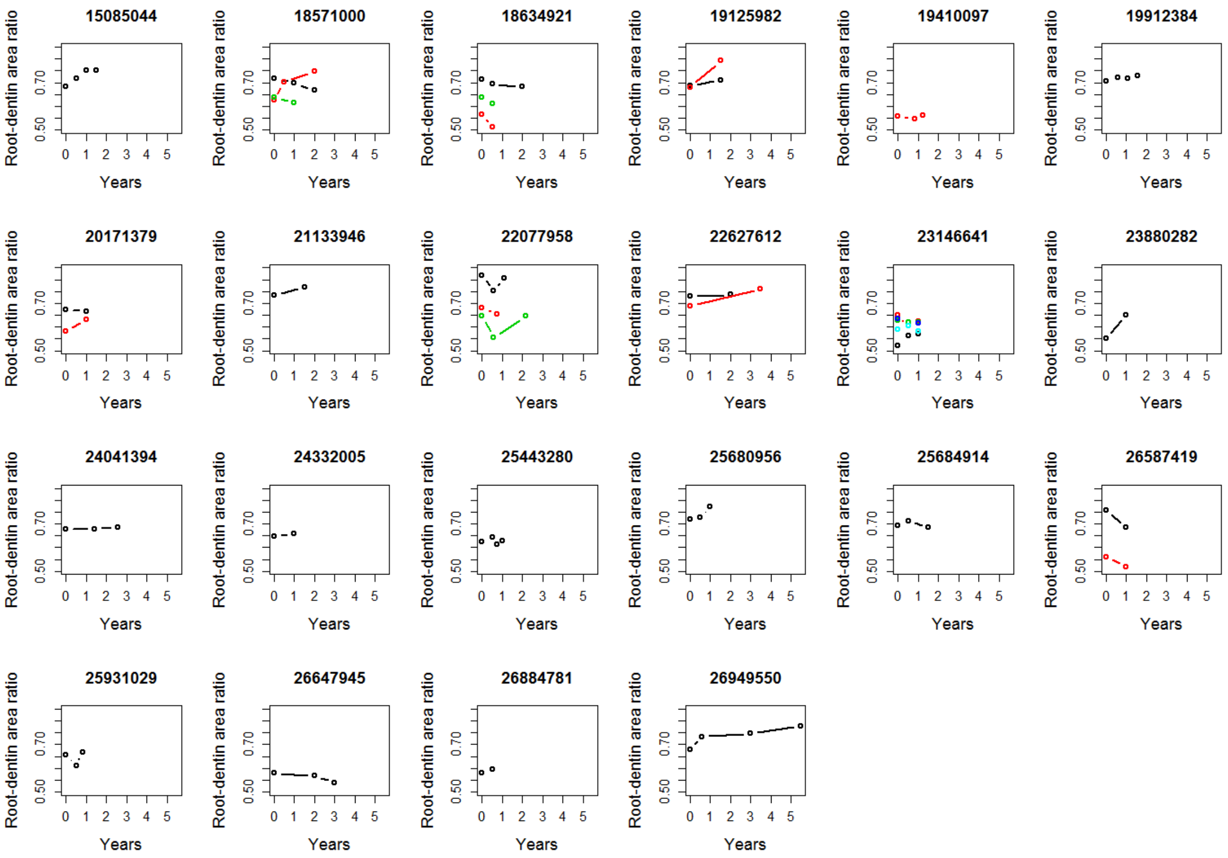

Supplemental Figure 3B.

Root-dentin area ratios plotted by author-graded apical closure types.

Root-dentin area was measured by subtracting total root-canal area below a line connecting two CEJs per tooth from the total mineralized root-dentin area. Root-dentin area ratios were calculated by dividing the root-dentin area against the total root area. Individual lines keyed to patients in Fig. 3.

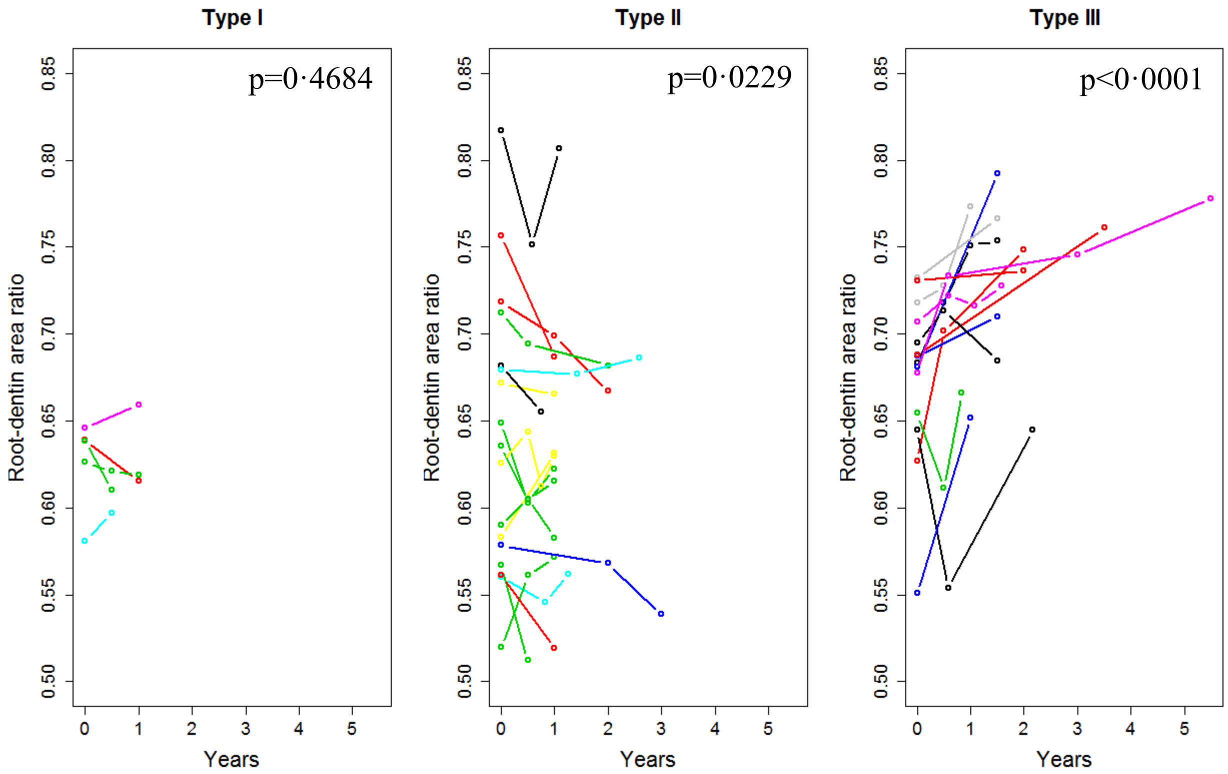

Supplement: Supplementary file 1 — Supplementary material [file 41598_2017_14412_MOESM1_ESM.pdf]
